# Energy Efficient Image/Video Data Transmission on Commercial Multi-Core Processors

**DOI:** 10.3390/s121114647

**Published:** 2012-11-01

**Authors:** Sungju Lee, Heegon Kim, Yongwha Chung, Daihee Park

**Affiliations:** Department of Computer Information Science, Korea University, Sejong KS002, Korea; E-Mails: peacfeel@korea.ac.kr (S.L.); khg86@korea.ac.kr (H.K.); dhpark@korea.ac.kr (D.P.)

**Keywords:** video sensor network, energy efficiency, multi-core processors

## Abstract

In transmitting image/video data over Video Sensor Networks (VSNs), energy consumption must be minimized while maintaining high image/video quality. Although image/video compression is well known for its efficiency and usefulness in VSNs, the excessive costs associated with encoding computation and complexity still hinder its adoption for practical use. However, it is anticipated that high-performance handheld multi-core devices will be used as VSN processing nodes in the near future. In this paper, we propose a way to improve the energy efficiency of image and video compression with multi-core processors while maintaining the image/video quality. We improve the compression efficiency at the algorithmic level or derive the optimal parameters for the combination of a machine and compression based on the tradeoff between the energy consumption and the image/video quality. Based on experimental results, we confirm that the proposed approach can improve the energy efficiency of the straightforward approach by a factor of 2∼5 without compromising image/video quality.

## 1. Introduction

In transmitting image/video data over Video Sensor Networks (VSNs), energy consumption must be minimized while maintaining high image/video quality [1]. Although image/video compression is well known for its efficiency and usefulness in VSNs, the excessive costs associated with the encoding computation and complexity still hinder its adoption in practical applications. Additionally, image/video compression techniques such as JPEG, JPEG2000, and H.264 [2–4] may degrade the image/video quality compared to the original image/video. However, it is anticipated that high-performance handheld multi-core devices will be used as processing nodes of VSNs in the near future, and the use of multi-core processors for handheld devices has been increasing. Since handheld devices operate with a battery, we need to consider energy consumption for efficiently compressing image/video content while still satisfying the user's image/video quality requirements. The use of multi-core processors is a possible way to not only reduce the execution time, but also improve the energy efficiency [5,6], thus parallel processing techniques using multi-core processors have become attractive for satisfying both real-time and energy efficiency requirements.

Parallel processing has been widely used to reduce the execution times of applications [5]. With advances in multi-core technology, multiprocessing techniques at a system software level have been used in order to reduce energy consumption [6]. However, parallel processing on multi-core processors may increase the total power consumption due to the use of more physical cores. Therefore, we need to evaluate the power-time tradeoff quantitatively.

Generally, there is a tradeoff between power consumption and execution time [7–11]. That is, if we increase the frequency (*i.e.*, processor speed), the power consumption is increased while the execution time is decreased. Because energy consumption is computed by a product of the power consumption and the execution time, we need to analyze the tradeoff with the given frequency.

Previous studies [7–11] conducted by the computer architecture community were targeted at designing general-purpose processors which could be applied to several applications. Processor vendors provide several levels of frequency settings and several numbers of cores, and it is the user's role to determine the optimal configuration for his/her application. Therefore, we need to optimize the system configuration at the software level (*i.e.*, the frequency setting and the number of cores) by analyzing the machine's characteristics and the application's parallelism collectively, because both the power consumption and the execution time depend on the number of cores and the application's parallelism.

To increase energy efficiency, compression techniques at the algorithmic level have been proposed [12–16]. Traditionally, many studies have been conducted to derive the optimal compression parameters using Rate-Distortion (R-D) analysis [12–14]. However, this traditional analysis has not considered the resource consumption of a platform, and may thus not be suitable for resource-constrained embedded devices or sensor network environments. Recently, some research results using Power-Rate-Distortion (P-R-D) analysis in order to control the power consumption of a network and maximize the video quality have been reported [15,16]. However, these analyses neither considered the compression time on the platform nor the machine's characteristics. Therefore, it is difficult to apply this analysis to an application's parallelism and energy efficiency when using a multi-core processor. Because of these difficulties, we need to analyze the characteristics of the machine and the compression collectively, and thus improve the energy efficiency of compression using a commercial multi-core processor.

In this paper, we propose Energy-Distortion (E-D) analysis in order to analyze the tradeoff between energy consumption of a platform and image/video quality in transmitting image/video data. In particular, we improve the energy efficiency of a commercial multi-core processor by using parallelism, because this analysis includes both the machine's and application's characteristics during the compression operation. Finally, we propose a general approach that can satisfy a user's requirements of image/video quality using E-D analysis.

In the experiments, we used three commercial multi-core processors (Intel quad-core i7and dual-core i5, AMD quad-core) [17,18] and analyzed the machines' characteristics. The energy efficiency was analyzed by measuring the actual power consumption with a WT210 power meter [19]. We also used three compression algorithms (JPEG, JPEG2000, and H.264), various image/video data, and diverse network conditions. Based on the experimental results with E-D analysis, the proposed approach can improve the energy efficiency of the straightforward approach by a factor of 2∼5 compared to the transmission of un-compressing/compressing data with equal image/video quality. We used a multi-core based notebook and did not consider the data capturing step since multi-core based sensor devices were not available to us during the experiments and our focus was only the compression and transmission step. Also, the battery consumption is proportional to the energy consumption, and although we could not measure the battery consumption directly, we believe that the proposed approach for energy efficiency can also extend the battery life of multi-core based sensor devices.

The rest of the paper is structured as follows: Section 2 describes the properties of commercial multi-core processors, the parallelism of applications, the multimedia compressions, and the control parameters. Section 3 explains the proposed approach for E-D analysis of machine characteristics and multimedia application characteristics, and the optimization of system configuration. Finally, Sections 4 and 5 describe the experimental results and conclusions, respectively.

## 2. Background

### 2.1. Commercial Multi-Core Processors

To improve the performance of computer systems, many studies related to the developments in semiconductor processes, distributed processing, and parallel processing technologies have been reported. With the advance of integrated circuit technology, the number of transistors and the frequency of processors have been improved significantly. However, improving the frequency is no longer possible due to high power consumption and heat dissipation, which should be reduced for resource-constrained, mobile/ubiquitous environments. To handle this issue, many hardware/software level studies have been reported [5–11].

Commercial multi-core processors have different characteristics according to the hardware architecture design. In Intel's multi-core architecture [17], the L2 cache is shared by two cores. In AMD's multi-core architecture [18], the L2 cache is allocated per core. According to service requirements, various hardware components (*i.e.*, memory, hard disk, IO devices, *etc.*) can be configured. Since the characteristics of the power consumption and execution time of the commercial multi-core processor depend on the design of the hardware architecture, it is difficult to generalize the power consumption and execution time characteristics. Therefore, to analyze the machine's characteristics, the power consumption and execution time need to be measured at least once.

### 2.2. Application's Parallelism

The execution time of an application on a multi-core processor depends on the application's parallelism. Amdahl's law provides a simple model to predict the speedup of parallel processing given the sequential portion of a program and the number of processors used.

Despite providing insight and usefulness, Amdahl's law considers neither the processor speed (*i.e.*, frequency) nor the power consumption. All the processor speeds are implicitly assumed to have the same (maximum) value. As the energy and the power are some of the most critical shared resources in a multicore-based parallel processor, it is not only interesting, but also necessary to collectively consider the implications of parallelization on the program performance and the energy consumption. Current technologies and design trends strongly indicate that future processors will be capable of *Dynamic Voltage and Frequency Scaling* (*DVFS* or *DVS* in short) [6]. Therefore, we need to collectively analyze the machine's characteristics (*i.e.*, the power and the execution time by setting the frequency and the number of cores) and the application's characteristics (*i.e.*, the application's parallelism), and thus improve the energy efficiency of applications using a commercial multi-core processor. Note that, we apply only the frequency scaling (without the voltage scaling) with the application level command, due to the limitations of our experimental environments.

### 2.3. Multimedia Compression

Generally, digital image/video data can be compressed using both lossy and lossless compression techniques. Lossy compression is a technique to remove spatial and temporal redundancy [2–4]. In image compression algorithms such as JPEG and JPEG2000, transformation coding (*i.e.*, discrete cosine transform and discrete wavelet transform) and quantization techniques have been studied in order to remove the spatial redundancy. Also, motion estimation and motion compensation have been studied in order to remove temporal redundancy between frames. Lossless compression such as Huffman coding and arithmetic coding is a technique to reduce the amount of statistical entropy.

**Figure 1.**
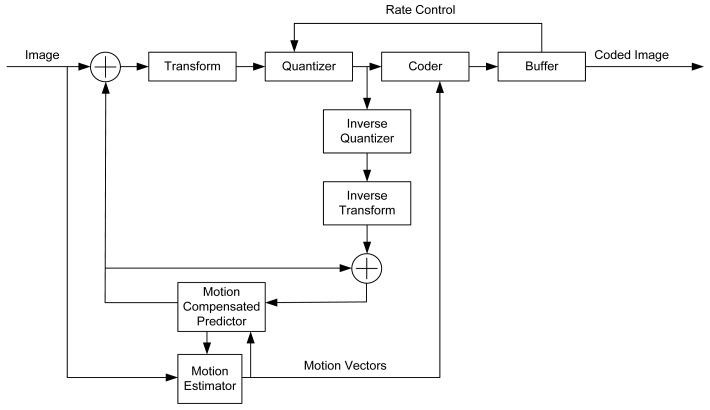
H.264 encoder [19].

JPEG and JPEG2000 are standards for still image compression. Notably, JPEG2000 has a rate-distortion advantage over JPEG. MPEG and H.264 are International Organization for Standardization (ISO) and International Telecommunication Union (ITU) standards for video compression. Figure 1 illustrates the H.264 video encoder.

Although image/video compression techniques can reduce the size of an original image/video, it may require more energy consumption due to the high computational complexity of the compression. Therefore, to reduce the energy consumption of image/video compression techniques, many studies using R-D analysis [12–14] or extended P-R-D analysis [15,16] have been reported.

### 2.4. Compression Control Parameters

In multimedia compression, the type of DCT, DWT, entropy coding and the size of the quantization table, etc., can be used as compression parameters. In this paper, we represent the compression parameter as *q* (*i.e.*, Quality Level of JPEG/JPEG2000, and Quality Parameter of H.264). The purpose of *q* is to control the compression rate and image/video quality with a scalable quantization table. *q* affects not only the image/video quality, but also lossless compression part (*i.e.*, entropy coding) after lossy compression (*i.e.*, DCT or DWT).

**Figure 2.**
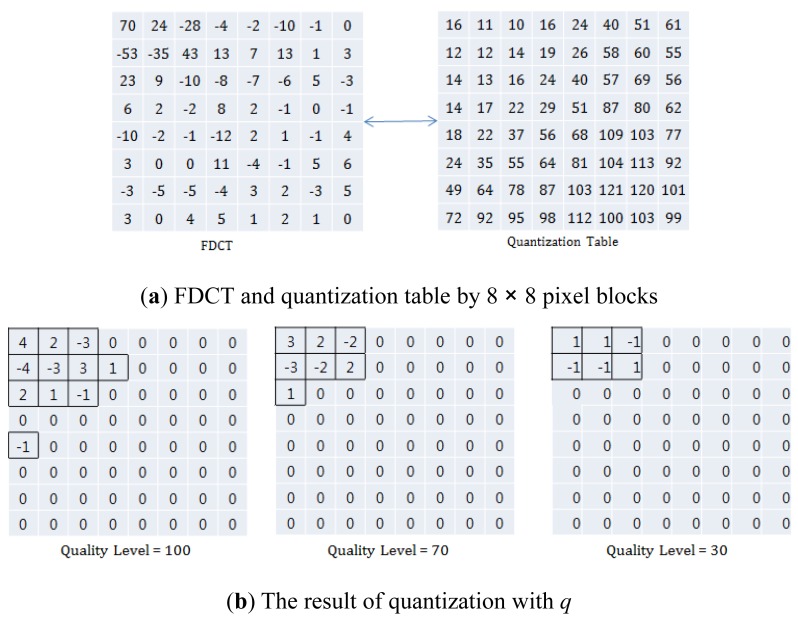
Illustration of *q* (*i.e.*, Quality Level or Quality Parameter).

In the compression procedure, the image/video is processed by 8×8 pixel blocks. Figure 2(a) shows an example of FDCT and Quantization Table by 8×8 pixel blocks. In Figure 2(b), the FDCT and Quantization Table results are calculated by (*FDCT_ij_/QuantizationTable*) × *q*/100, where *q =* 1, 2, …, 99, 100. Since the number of zeros is increased with decreased *q*, the computation of lossless compression and the compressed image/video size are decreased, and the image/video quality is also decreased. Note that, the computation of lossless compression is maximized where *q* = 100, and also the image/video quality is maximized. In contrast, the computation of lossless compression is minimized where *q*=1, and also the image/video quality is minimized. Therefore, we can control the amount of computation, compression rate, and image/video quality with *q* [2–4].

## 3. Proposed Approach

We propose an experiment-based model in order to evaluate the performance of a given application on a machine collectively. We measure the power consumption of a test application “only once” with every combination of the number of cores and frequency of a machine in order to understand the *machine*'s characteristics. Then, we measure the execution time of a given application only with the single core and at maximum frequency of a machine in order to understand the *application*'s characteristics. With these two measurements, we can estimate the energy performance of the given application with “any” combination of the number of cores and frequency of the machine. Also, we propose a greedy approach to find the optimal parameters for the energy efficiency in transmitting image/video data without compromising image/video quality.

### 3.1. Machine's and Application's Characteristics

First, to understand the machine's and application's characteristics, we measured the power consumption, execution time, and the energy consumption of parallelized AES-CBC (*i.e.*, 0% parallelism), AES-CCM (*i.e.*, 50% parallelism) and AES-CTR (*i.e.*, 100% parallelism) [21] with the *Pthread* library [20] as examples of test applications on the Intel i7 and AMD multi-core processors. The AES-CTR problem has no data dependency and is easily parallelized. In contrast, AES-CCM has 50% data dependency, and AES-CBC has 100% data dependency. According to Amdahl's law, the maximum speedup (with a 4-core processor) of AES-CTR and AES-CCM are 4 and 2, respectively. Note that AES-CCM combines encryption and authentication, and it is widely used in wireless applications.

**Figure 3.**
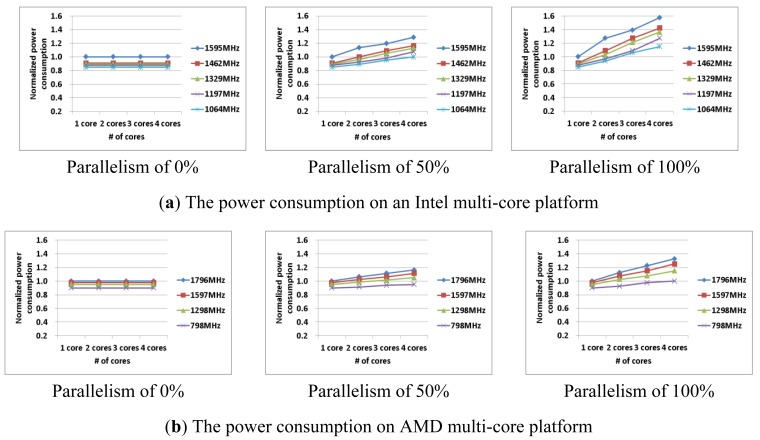
The power consumption with various test an applications on multi-core platforms.

Figure 3 shows the power consumption and execution time of the test applications with 0%, 50%, 100% parallelism on multi-core processors, with various frequencies and numbers of cores. The power consumption, the execution time, and the energy consumption were normalized based on the case with a single core and maximum frequency. As shown in Figure 3, the power consumption increased and execution time decreased with increased frequency and number of cores. In the results, it can be seen that these characteristics have similar patterns for each processor. Since increasing or decreasing rates of power consumption and execution time are different across processors, the power consumption and execution time of a processor should be measured at least once in order to analyze the processor's characteristics. As shown in Figure 3, we found that applications with less parallelism can use fewer cores, and thus less power is consumed.

**Figure 4.**
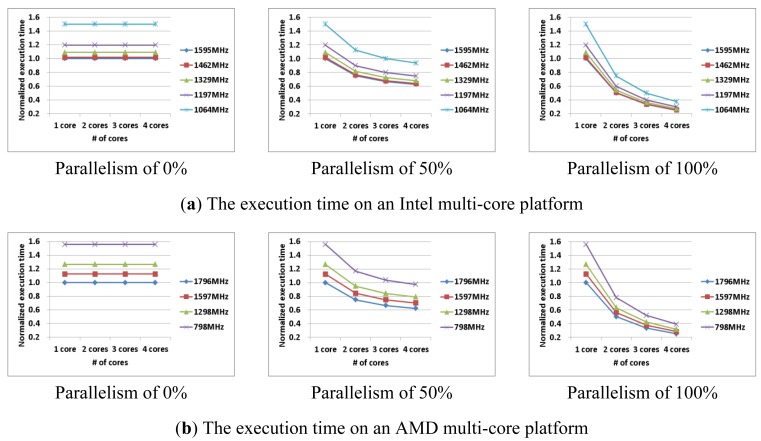
The execution time with test applications on multi-core platforms.

Although an application with less parallelism requires less power consumption, it may consume more energy due to greater execution time. Figure 4 shows the execution time of AES-CBC, AES-CCM, and AES-CTR on 1, 2, 3, and 4 cores. AES-CBC (0% parallelism) can be performed with increased number of the cores, but both the power consumption and the execution time are always constant (see Figures 3 and 4). In contrast, as we increase the number of cores in AES-CTR (100% parallelism), the execution time decreases while the power consumption increases. To improve the energy efficiency, we need a collective analysis of the machine and application characteristics.

**Figure 5.**
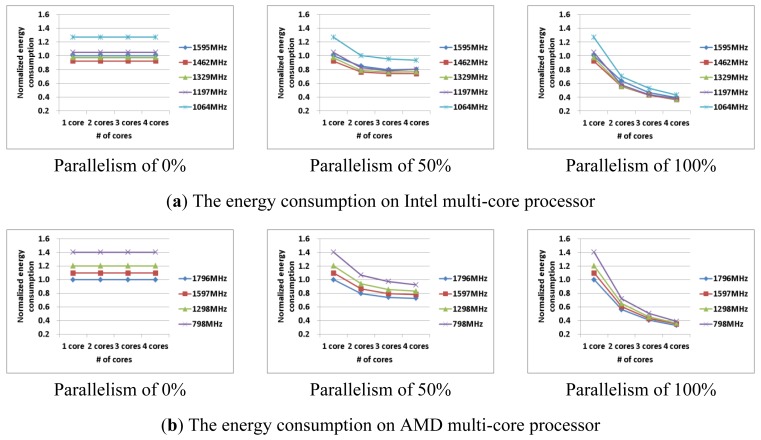
The energy consumption with test applications on multi-core processors.

Figure 5 shows the energy consumption with various parallel applications on Intel and AMD processors. On the Intel processor, the optimal frequency is always 1,462 MHz, but each optimal number of cores is different for each amount of parallelism: one core (0% parallelism), three cores (50% parallelism), and four cores (100% parallelism). On the AMD processor, the optimal frequency is always 1,796 MHz, and the optimal number of cores is also different for different amounts of parallelism: one core (0% parallelism), four cores (50% parallelism), and four cores (100% parallelism). In this paper, we propose a way to improve the energy efficiency by using optimal machine parameters (*i.e.*, the frequency and the number of cores) according to application's parallelism. We generated a performance metric for the power consumption in order to understand the machine's characteristics, and then predicted the energy consumption by an application's parallelism using Amdahl's law.

### 3.2. Collective Analysis of Machine's and Application's Characteristics

First, we analyze the relationship between the application/machine and the energy consumption. The power consumption and the execution time depend on the characteristics of the machine and the application. Thus, we can represent the energy consumption *E* by Equation (1) with power consumption *W* and execution time *T*:

(1) E=W×T

**Figure 6.**
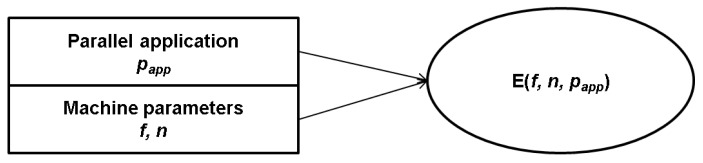
The relationship between application/machine characteristics and the energy consumption.

To analyze the power consumption and the execution time with an application's parallelism, we denote the application's parallelism as *p_app_*, where 0 ≤ *p_app_* ≤ 1.The application's parallelism (*i.e.*, *p_app_*), frequency (*i.e.*, *f*), and number of cores (*i.e.*, *n*) sensitively affect the energy consumption of a processor as shown in Figure 6. Thus, the energy consumption is represented as Equation (2), where *f* is the frequency and *n* is the number of cores. To reduce the energy consumption, we need to set the optimal *f* and *n* with a prediction of the energy consumption from the given application and machine characteristics.

(2) $$E(f,n,p_{\text{app}})=W(f,n,p_{\text{app}})\times T(f,n,p_{\text{app}})$$

The power consumption can be measured with an application having 100% parallelism (*i.e.*, AES-CTR). With an increased number of cores, the power consumption is also increased. We can also find that the power consumption depends on the number of cores. Thus, when the combination of application and machine characteristics are given, we can analyze the application's parallelism. We can predict the power consumption by using Equation (3) with the measured results. We focus only on the dynamic power consumption of the whole multi-core based platform at the compression and transmission step although the static power consumption at the idle time is not negligible.

Note that, the power varies during the execution of the given application. We measured the power consumption at several points and took the average. For simplicity, we used this average value as the power consumption value. Note also that, an application consists of a sequential portion (having some data dependency) and a parallel portion (not having any data dependency). We denote the power consumption of the sequential portion of the application with *1* core as *W_sequential_*(*f, 1*) and the power consumption of the parallel portion of the application with *n* cores *W_parallel_* (*f, n*). As shown in Figure 3 (with the 0% parallelism case), the power consumption of the sequential portion of the application is independent with the number of cores. Therefore, *W_sequential_*(*f, 1*) = *W_sequential_*(*f, n*) (*i.e.*, the power consumption of the sequential portion of the application with *n* cores).

(3) $$W(f,n,p_{\text{app}})\approx W_{\text{sequential}}(f,1)\times (1-p_{\text{app}})+W_{\text{parallel}}(f,n)\times (p_{\text{app}})$$

Also, the total execution time (*i.e.*, *T*(*f*, *n*, *p_app_*), with various numbers of cores can be predicted using Equation (4). *W_sequential_*(*f, 1*) and *T_sequential_*(*f, 1*) represent the power consumption and the execution time of the sequential portion of the application, respectively. As shown in Figures 3 and 4 (parallelism of 0% case), both *W_sequential_*(*f, 1*) and *T_sequential_*(*f, 1*) are independent with the number of cores. In contrast, *W_parallel_*(*f, n*) and *T_parallel_*(*f, n*) represent the power consumption and the execution time of the parallel portion of the application, respectively. As shown in Figures 3 and 4 (parallelism of 100% case), both *W_parallel_*(*f, n*) and *T_parallel_*(*f, n*) depend on the number of cores.

We denote the execution time of the sequential portion of the application with *1* core as *T_sequential_*(*f, 1*) and the execution time of the parallel portion of the application with *n* cores *T_parallel_*(*f, n*). As shown in Figure 4 (with the 0% parallelism case), the execution time of the sequential portion of the application is independent with the number of cores. Therefore, *T_sequential_*(*f, 1*) = *T_sequential_*(*f, n*) (*i.e.*, the execution time of the sequential portion of the application with *n* cores). Note that, if we denote the execution time of the parallel portion of the application with *1* core as *T_parallel_* (*f, 1*), then *T_parallel_* (*f, n*) is not equal to *T_parallel_* (*f, 1*)*/n* in a strict sense, due to the pthread overhead. However, *T_parallel_* (*f, n*) can be approximately equal to *T_parallel_* (*f, 1*)*/n*, with a careful parallelization:

(4) $$T(f,np_{\text{app}})\approx T_{\text{sequential}}(f,1)\times (1-p_{\text{app}})+T_{\text{parallel}}(f,1)/n\times (p_{\text{app}})$$

### 3.3. E-D Analysis

In general, to control the compression rate and image/video quality, compression parameters are widely used by the multimedia compression community. Recently, to improve the energy efficiency, Rate-Distortion (R-D) and Power-Rate-Distortion (P-R-D) analysis have been reported [15,16]. In this paper, we propose E-D analysis in order to analyze the energy efficiency of the machine and the required image/video quality collectively.

**Figure 7.**
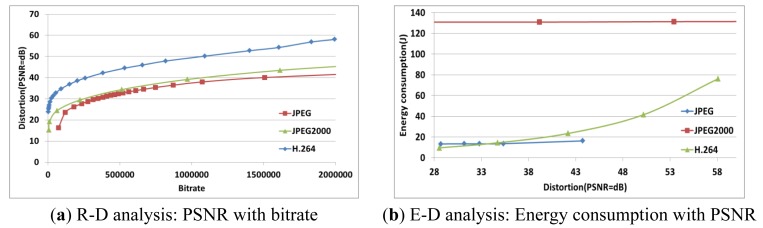
Comparison of performance with JPEG, JPEG2000, and H.264.

R-D or P-R-D analysis is not enough to evaluate multimedia compression algorithms such as JPEG, JPEG2000, and H.264 in terms of the energy consumption and image/video quality. However, the proposed E-D analysis can evaluate them. Figure 7 compares the performance of JPEG, JPEG2000, and H.264.

With E-D analysis, the energy consumption to compress/transmit the multimedia data *E_comp+trans_* is represented as Equation (5):

(5) $$E_{\text{comp}+\text{trans}}=E_{\text{comp}}+E_{\text{trans}}$$

The image/video quality (*i.e.*, distortion) is represented as Equation (6), where PSNR (*i.e.*, peak signal to noise ratio) is widely used as a performance indicator to evaluate image/video distortion by the multimedia compression community. In this paper, we represent the compression parameter as *q* (*i.e.*, Quality Level of JPEG, JPEG2000, and Quality Parameter of H.264). The purpose of *q* is to control the compression rate and image/video quality with a scalable quantization table:

(6) D(q)=PSNR

**Figure 8.**
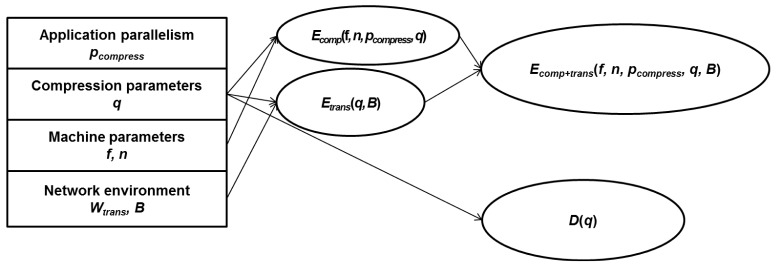
The relationship between the energy consumption and the image/video quality.

Figure 8 shows the energy consumption and the image/video quality with the *q* parameter. We found that *q* affects both the compression energy consumption and the transmission energy consumption. To minimize the total energy consumption, we need collective analysis that considers machine and application characteristics.

**Figure 9.**
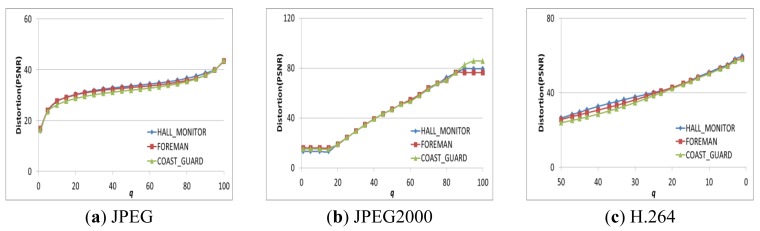
PSNR with *q*.

To analyze the energy consumption and image/video quality by controlling *q,* we can find the image/video quality (*i.e.*, PSNR) with *q* as shown in Figure 9. Specifically, we use three types of multimedia data (HALL_MONITOR, FOREMAN, and COAST_GUARD) of CIF size, and three compression algorithms (JPEG, JPEG2000, and H.264). The image/video quality of each compression algorithm is similar to *q*. Thus, controlling *q* is a possible way to satisfy a user's image/video quality requirements.

**Figure 10.**
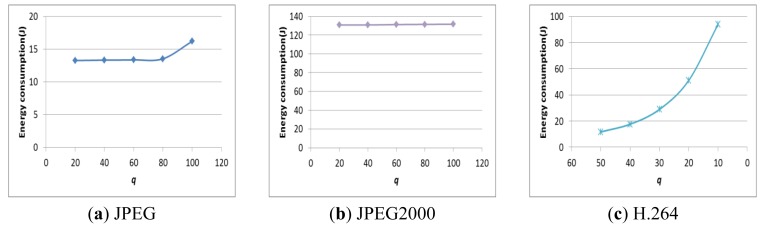
The energy consumption with *q*.

Figure 10 shows the total energy consumption with *q*. In fact, the power consumption may not be affected by *q*, but the execution time depends on *q*. Therefore, *q* should be determined in order to improve the energy efficiency by using the E-D analysis while satisfying the user's image requirements.

**Figure 11.**
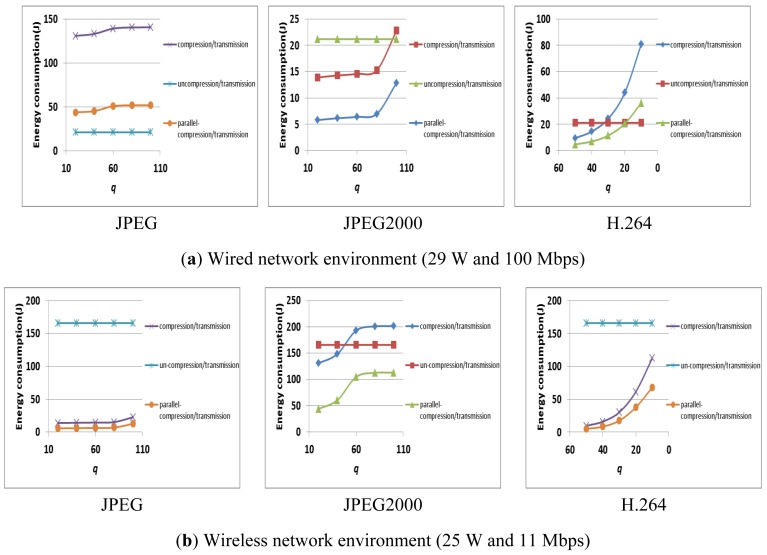
E-D anal$ysis on commercial multi-core processors in various network environments.

Figure 11 shows the result of the E-D analysis on a commercial multi-core platform (*i.e.*, Intel i7 quad-core processors) in different network environments (*i.e.*, a wired network that supports 100 Mbps with 15 W, and a wireless network that supports 11 Mbps with 11 W). As shown in Figure 11, the energy consumption of compression/transmission depends on the machines, the parallelism of the applications, and the network environment.

This is because the compression computation affects the machine's energy consumption, and both the compression ratio and the transmission bandwidth affect the transmission's energy consumption. Also, in these given environments (*i.e.*, the machines, the parallelism of the applications, the network environment), we should determine whether the compression is applied or not. For example, in Figure 11(a) with JPEG and a wired network, the un-compression/transmission case is always better than the compression/transmission case. However, parallel-compression/transmission using 4 cores can reduce the energy consumption of the un-compression/transmission. Also, in Figure 11(b) with JPEG and a wireless network, both the compression/transmission and the parallel-compression/transmission are always better than the un-compression/transmission. Therefore, given these environments (*i.e.*, commercial multi-core platforms and compression algorithms), we should select the compression/transmission, the parallel-compression/transmission, or the un-compression/ transmission by using the E-D analysis.

### 3.4. Optimization of System Configuration

In this paper, we propose a greedy approach to find the optimal parameters for the energy efficiency in transmitting image/video data without compromising image/video quality. Algorithm 1 shows the procedure to find the optimal frequency *f* and the number of cores *n* by using a greedy approach.

**Algorithm 1.** Finding Optimal Machine Parameters.given the environment parameter *p_app_*← application's parallelismset the default parameters *f* ← maximum frequency *n* ← 1 coredo { calculate *E*(*f, n, p_app_*) if (*n_next* is not last level) {  *n_next* ← next increased level  calculate *E*(*f, n_next, p_app_*)} if (*f_next* is not last level) {  *f_next*← next decreased level  calculate *E*(*f_next, n, p_app_*)} if (*E*(*f, n_next, p_app_*)<*E*(*f, n, p_app_*)) *n*←*n_next* if (*E*(*f_next, n, p_app_*)<*E*(*f, n_next, p_app_*)) *f*←*f_next*} while ((*E*(*f, n, p_app_*)<*E*(*f, n_next, p_app_*) AND *E*(*f, n, p_app_*)<*E*(*f_next, n, p_app_*))*f_opt*←*f //* found optimal frequency*n_opt*←*n//* found optimal cores

Note that *p_app_* is a given parameter which can be gained by application parallelism. The energy consumption can be represented as Equation (7), which consists of compression energy *E_comp_* and transmission energy *E_trans_*. *E_comp_* is represented by a compression parameter *q* as in Equation (7):

(7) $$E_{\text{comp}}(q)=W_{\text{comp}}(q)\times T_{\text{comp}}(q)$$

Since the compression energy consumption should be considered for the given machine and parallel application, *E_comp_* is represented as in Equation (8). *D*(*q*) is the image/video quality with compression parameters, and *D_0_* (*i.e.*, PSNR) is the user's requirement of image/video quality:

(8) $$E_{\text{comp}}(f,n,p_{\text{compress}},q)=W_{\text{comp}}(f,n,p_{\text{compress}},q)\times T_{\text{comp}}(f,n,p_{\text{compress}},q)$$

We also need to analyze the transmission energy consumption to minimize the total energy consumption. The transmission energy consumption *E_trans_* is represented as Equation (9):

(9) $$E_{\text{trans}}=W_{\text{trans}}\times T_{\text{trans}}$$

The machine, network environment, and compression rate affect the transmission energy consumption. Thus, the transmission energy consumption is represented as Equation (10). *M* is the compressed data size determined by the compression parameter (*i.e.*, *q*), and *B* is the network bandwidth (*i.e.*, unit: bit per second).

(10) $$E_{\text{trans}}(q,B)=W_{\text{trans}}\times M(q)/B$$

By using Equations (6) and (11) collectively, we can minimize the total energy consumption *E_comp+trans_* while satisfying the user's image/video quality requirements:

(11) $$\begin{array}{cc} \text{min}E_{\text{comp}+\text{trans}}(f,n,p_{\text{compress}},q,B)=\min[E_{\text{comp}}(f,n,p_{\text{compress}},q)+E_{\text{trans}}(q,B)] & s.t.D(q)>D \end{array}$$

Finally, we can find the optimal compression and machine parameters (*i.e.*, the frequency *f* and the number of cores *n*) by using Algorithm 2.

**Algorithm 2.** Finding Optimal Machine and Compression Parameters.given environment parameters *p_compress_*← compression application's parallelism *B* ← network bandwidth *D_0_*← user's requirement for image/video qualityfind machine's parameters by using algorithm 1 *f*←*f_opt* *n*←*n_opt*set the default compress parameter *q* ← maximum image/video quality parameterdo{ calculate *E_comp+trans_*(*f, n, p_compress_, q, B*) *q_next*← next decreased image/video quality parameter calculate *E_comp+trans_*(*f, n, p_compress_, q_next, B*) if (*E_comp+trans_*(*f, n, p_compress_, q_next, B*)*<E_comp+trans_*(*f, n, p_compress_, q, B*))  *q*←*q_next*} while (*D*(*q*) >*D_0_*)*q_opt*←*q* // found optimal compress parameter

In addition, we can select the compression/transmission, the parallel-compression/transmission, or the un-compression/transmission scenario by using Algorithm 3.

**Algorithm 3.** Selection of the Minimum Energy Consumption Scenario.given environment parameters *p_compress_*← compression application's parallelism *B* ← network bandwidthset the optimal parameters by using algorithm 1 and 2 *f*←*f_opt* *n*←*n_opt* *q* ← *q_opt*if (*E_trans_*(*no_compress*) <*E_comp+trans_*(*f, n, p_compress_, q, B*)) select *E_trans_*(*no_compress*)else select *E_comp+trans_*(*f, n, p_compress_, q, B*)

## 4. Experimental Results

We present the experimental results. The experimental environment is described in Section 4.1. Then, the energy efficiency that results from using the E-D analysis is explained in Section 4.2.

### 4.1. Experimental Environments

**Figure 12.**
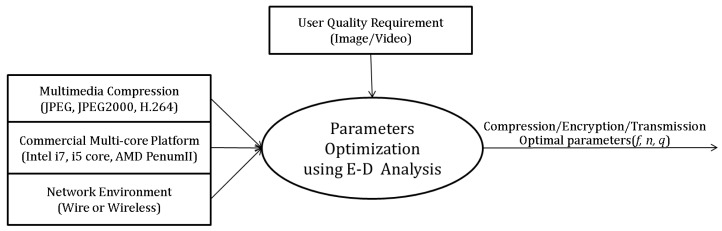
The experimental environment.

To evaluate the energy efficiency that results from using the E-D analysis, we configured the experimental environment as shown in Figure 12.

We used three commercial multi-core platforms (*i.e.*, Intel quad-core i7 and dual-core i5, AMD quad-core), which are summarized in Table 1.

We configured the network environment as wired (100 Mbps) and wireless (11 Mbps). Table 2 shows the power consumption of the network devices on the i7, i5, and AMD platforms, respectively.

**Figure 13.**
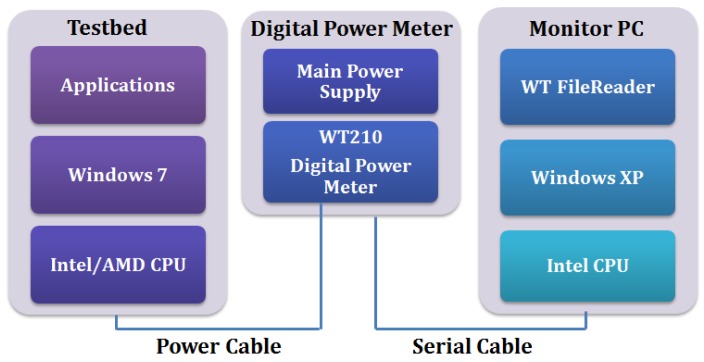
Configuration of the power measurement environment.

Figure 13 shows the configuration of the measurement environment. We measured the actual power consumption using a WT210 power meter [19]. We considered the power consumption of the whole system at the compression/transmission step with various machine and application parameters.

**Figure 14.**
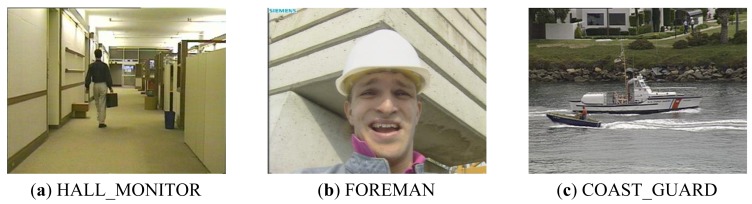
Image/Video data set [22].

We used three compression algorithms (*i.e.*, JPEG, JPEG2000, and H.264), and various image/video data. For parallel compression algorithms, we parallelized JPEG, JPEG2000 with Pthread [20], and used parallel H.264 of the PARSEC benchmark suite [23]. We selected CIF-size HALL_MONITOR, FORMAN, and COAST_GUARD from the image/video data set [22], and Figure 14 shows these input data.

### 4.2. Experimental Analysis

#### 4.2.1. Accuracy Validation of Prediction Parameters

First, to evaluate the prediction accuracy, we measured the performance of AES-CCM with 100% parallelism on each machine. Tables 3–5 show the normalized energy consumption of each machine. With these results, we can predict the energy consumption and find the optimal frequency and number of cores. We normalized the power consumption, execution time, and energy consumption based on a single core and the maximum frequency, and the user's image/video quality requirements.

We also analyzed the parallelism of JPEG, JPEG2000, and H.264 applications, which were 0.97, 0.95, and 0.93, respectively. With the parallelism analyzed, we can predict the normalized energy consumption, and find the machine parameters (*i.e.*, frequency *f* and number of cores *n*). Table 6 shows the estimated and measured results from the energy consumption analysis.

Table 7 shows the estimated and measured results from E-D analysis on i7, i5, and AMD platforms on wired/wireless networks (*i.e.*, 100 Mbps and 11 Mbps), with a quality requirements of PSNR > 30 dB. Based on the results, we confirmed that our prediction of energy consumption is accurate and can determine the optimal machine and compression parameters to improve the energy efficiency while satisfying quality requirements. Finally, we can select the minimum energy consumption scenario with the comparison of E-D analysis and un-compress scenario.

#### 4.2.2. Results from E-D Analysis

To evaluate the energy efficiency that results from using the E-D analysis, we compared several scenarios and the proposed approach as shown in Table 8. The baseline scenarios 1-A and 1-B are for the un-compression/transmission case and the compression/transmission case, respectively. In scenario 1, we examine the frequency as a single core and maximum frequency. Also, we set the *q* parameter as 25 (*i.e.*, H.264) or 50 (*i.e.*, JPEG and JPEG2000). The scenarios 2 and 3 are for the computer architectural approach and the multimedia compression approach, respectively. In scenario 2, we set the optimal machine parameters (*i.e.*, frequency and the number of cores), and the compression parameter (*i.e.*, *q*) as 25 or 50. In scenario 3, we set the optimal compression parameters, and used the maximum frequency and 1 core. Finally, in scenario 4, we set the optimal machine and compression parameters collectively by using the E-D analysis.

Scenario 4 is a way to improve the energy efficiency with both the machine and multimedia compression parameters collectively. Table 9 shows the results of the optimal machine and multimedia compression parameters.

**Figure 15.**
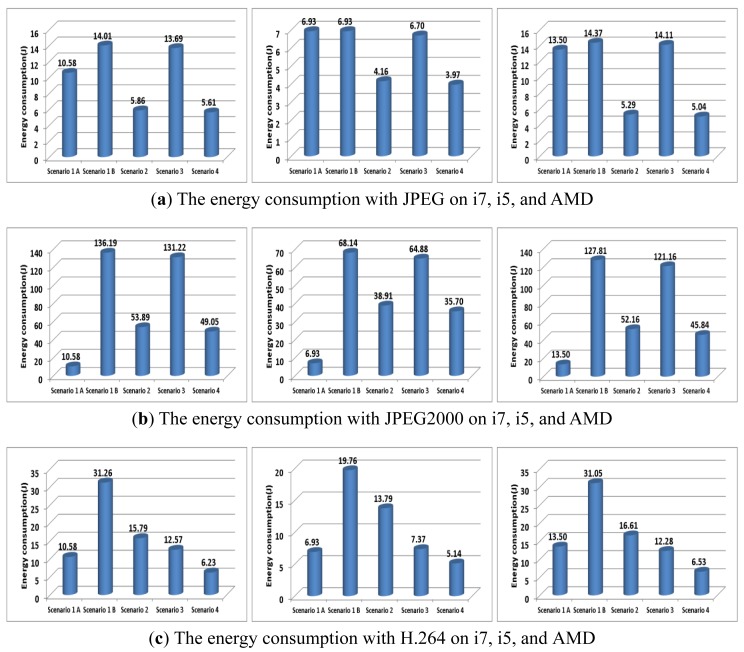
The energy consumption with various scenarios over wired network.

**Figure 16.**
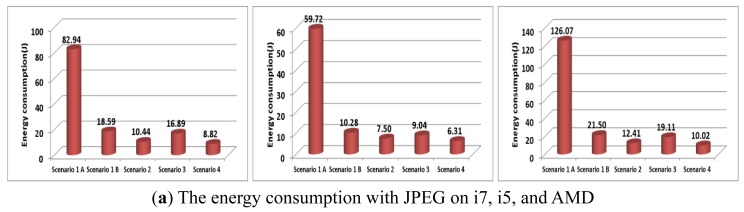

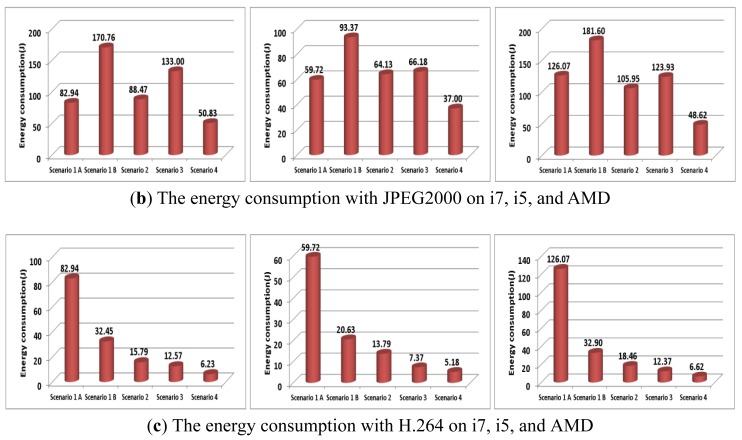
The energy consumption with various scenarios over wireless network. (**a**) The energy consumption with JPEG on i7, i5, and AMD (**b**) The energy consumption with JPEG2000 on i7, i5, and AMD (**c**) The energy consumption with H.264 on i7, i5, and AMD

Finally, the scenarios 1, 2, 3, and 4 on each machine are shown in Figures 15 and 16. In the given environments, scenario 4 (*i.e.*, E-D analysis) can provide the minimum energy consumption. The wireless network consumed more energy than the wired network. With JPEG2000 in the wired network environment shown in Figure 15(b), the energy consumption of scenario 1-A (*i.e.*, un-compression) was less than that in scenarios 2, 3, and 4. However, scenario 4 can provide the minimum energy consumption with the wireless network, as shown in Figure 16(b). Since the energy consumption of H.264 is more affected by the multimedia compression parameters than the machine parameters, scenario 3 consumed less energy than scenario 2. However, scenario 4 can provide the minimum energy consumption, regardless of the network. Therefore, in the given environments, we can improve the energy consumption by using E-D analysis for a given image/video quality.

**Figure 17.**
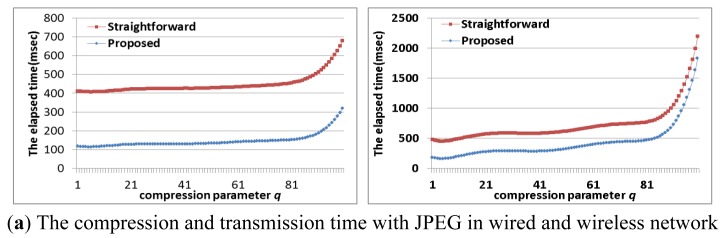

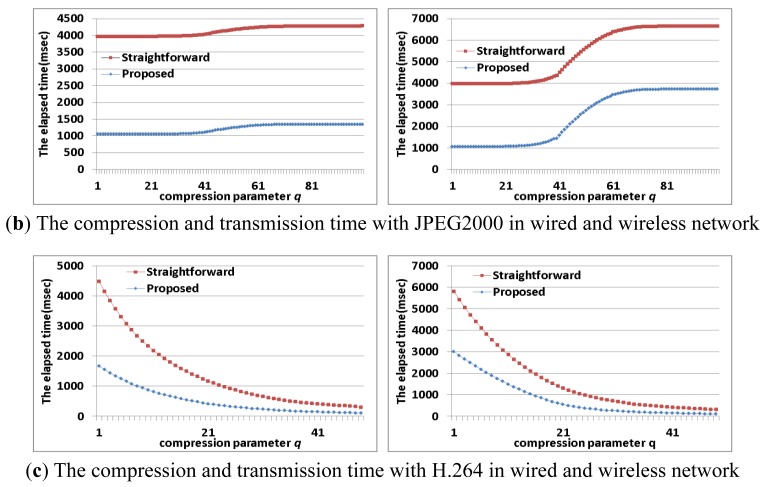
The elapsed time with JPEG/JPEG2000/H.264 in wired and wireless network.

We focused on reducing the energy consumption at the compression/transmission step by using multi-core based sensor nodes. However, the latency at the compression/transmission step is also important. In order to evaluate the effect of the proposed approach (*i.e.*, scenarios 4 in Table 8: the optimal number of cores with the optimal frequency and the optimal compression parameter) on the latency, we compared the elapsed time at the compression/transmission step. As shown in Figure 17, the proposed approach can also reduce the elapsed time of the straightforward approach (*i.e.*, scenarios 1-B in Table 8: single core with the maximum frequency and the default compression parameter).

## 4. Conclusions

Multi-core processors have been used recently for embedded systems, in addition to PCs and servers. Therefore, many studies have been conducted in order to apply commercial multi-core processors to real applications. This paper proposed an approach that could provide both high energy efficiency and high image/video quality by analyzing machine and application characteristics collectively. From the given multi-core platform and network environment, the proposed approach can provide a collective analysis by considering both machine and application characteristics. We proposed E-D analysis in order to analyze the tradeoff between energy consumption of a platform and image/video quality. In particular, we improved the energy efficiency of a commercial multi-core platform by using parallelism because this analysis includes both the machine's characteristics and the application's characteristics during the compression operation. Based on the experimental results with image/video data and Pthread programming model, the proposed approach with E-D analysis can improve the energy efficiency of typical approaches used by computer architecture or multimedia compression communities by a factor of 2∼5 with equal multimedia quality. We believe the proposed approach can be applied to real scenarios such as VSNs with multi-core processors in the near future.

## Figures and Tables

**Table 1.** Platforms specs. of Intel i7 and i5, AMD processors.

		**i7**	**i5**	**AMD**
Processor		Intel i7 720QM	Intel i5 core	AMD PenumII
Frequency range		1.0 GHz∼1.5 GHz	0.9 GHz∼1.5 GHz	0.7G Hz∼1.7 GHz
Frequency step		133 MHz	100 MHz	500/300/200 MHz
The maximum # of cores		4	2	4
Network device	Wired	Intel(R) 82577LM Gigabit Network Connection	RealtekPCIe GBE Family Controller	JMicron PCI Express Gigabit Ethernet Adapter
	Wireless	Intel(R) Centrino(R) Advanced-N 6200 AGN	Broadcom 802.11n Network Adapter	Athreos AR9285 Wireless Network Adapter

**Table 2.** Power consumption of the network devices on i7, i5, and AMD platforms.

	**i7**	**i5**	**AMD**
Wired (100 Mbps)	28.5 W	17.0 W	37.5 W
Wireless (11 Mbps)	24.5 W	19.0 W	38.5 W

**Table 3.** Normalized energy consumption on i7 platform.

Actual	i7			
	1 core	2 cores	3 cores	4 cores
1,595MHz	100%	63%	49%	41%
1,462MHz	99%	59%	47%	39%
1,329MHz	108%	61%	47%	41%
1,197MHz	117%	65%	50%	41%
1,064MHz	131%	71%	53%	44%

**Table 4.** Normalized energy consumption on i5 platform.

Actual	i5	
	1 core	2 cores
1,397MHz	100%	55%
1,297MHz	106%	57%
1,197MHz	115%	62%
1,097MHz	123%	66%
997MHz	136%	74%

**Table 5.** Normalized energy consumption on AMD platform.

Actual	AMD			
	1 core	2 cores	3 cores	4 cores
1,796MHz	100%	56%	43%	34%
1,597MHz	107%	61%	45%	37%
1,298MHz	176%	92%	67%	54%
798MHz	210%	107%	75%	60%

**Table 6.** The estimated and measured results from the energy consumption analysis.

	JPEG		JPEG2000		H.264	
	*p_compress_* = 0.97		*p_compress_* = 0.95		*p_compress_* = 0.93	
	Estimated	Measured	Estimated	Measured	Estimated	Measured
i7	1462, 4 (MHz, # of cores)	1462, 4 (MHz, # of cores)	1462, 4 (MHz, # of cores)	1462, 4 (MHz, # of cores)	1462, 4 (MHz, # of cores)	1462, 4 (MHz, # of cores)
	42%	39%	44%	40%	46%	38%
i5	1397, 2 (MHz, # of cores)	1397, 2 (MHz, # of cores)	1397, 2 (MHz, # of cores)	1397, 2 (MHz, # of cores)	1397, 2 (MHz, # of cores)	1397, 2 (MHz, # of cores)
	56%	57%	57%	59%	58%	59%
AMD	1796, 4 (MHz, # of cores)	1796, 4 (MHz, # of cores)	1796, 4 (MHz, # of cores)	1796, 4 (MHz, # of cores)	1796, 4 (MHz, # of cores)	1796, 4 (MHz, # of cores)
	36%	33%	38%	35%	40%	35%

**Table 7.** The estimated and measured results from E-D analysis on i7, i5, and AMD platforms.

		Machine Parameters *f, n* (MHz, # of cores)	Compression Parameters *q Distortion*(*q*) *> 30 dB*	Normalized energy consumption (wired/wireless)
				E-D analysis
i7				
JPEG	Estimated	1462, 4	17	43%/60%
	Measured	1462, 4	20	44%/63%
JPG2000	Estimated	1462, 4	31	39%/39%
	Measured	1462, 4	33	39%/39%
H.264	Estimated	1462, 4	44	15%/14%
	Measured	1462, 4	37	18%/19%
i5				
JPEG	Estimated	1397, 2	17	63%/91%
	Measured	1397, 2	20	63%/91%
JPG2000	Estimated	1397, 2	31	55%/57%
	Measured	1397, 2	33	55%/58%
H.264	Estimated	1397, 2	44	11%/9%
	Measured	1397, 2	37	12%/10%
AMD				
JPEG	Estimated	1796, 4	17	37%/98%
	Measured	1796, 4	20	38%/98%
JPG2000	Estimated	1796, 4	31	39%/46%
	Measured	1796, 4	33	41%/67%
H.264	Estimated	1796, 4	44	4%/3%
	Measured	1796, 4	37	6%/4%

**Table 8.** Scenarios of the image/video transmission.

	**Machine Parameters**		**Compression Parameter *q***
	**Frequency**	**# of cores**	
Scenario 1-A. BASELINE Un-compression and Transmission	Maximum	1core	-
Scenario 1-B. BASELINE Compression and Transmission	Maximum	1core	25 (H.264) or 50 (JPEG/JPEG2000)
Scenario 2 Computer Architectural Approach	Optimum	Optimum	25 (H.264) or 50 (JPEG/JPEG2000)
Scenario 3 Multimedia Compression Approach	Maximum	1core	Optimum
Scenario 4 Optimization with E-D Analysis	Optimum	Optimum	Optimum

**Table 9.** The optimal machines and multimedia compression parameters.

		**i7**	**i5**	**AMD**
JPEG	Frequency *f*	1,462 MHz	1,397 MHz	1,796 MHz
	# of cores *n*	4	2	4
	Compress parameter *q* PSNR = 30.22 dB	17	17	17
JPEG2000	Frequency *f*	1,462 MHz	1,397 MHz	1,796 MHz
	# of cores *n*	4	2	4
	Compress parameter *q* PSNR = 30.22 dB	31	31	31
H.264	Frequency *f*	1,462 MHz	1,397 MHz	1,796 MHz
	# of cores *n*	4	2	4
	Compress parameter *q* PSNR = 30.22 dB	44	44	44
